# Pituitary metastasis of hepatocellular carcinoma presenting with panhypopituitarism: a case report

**DOI:** 10.1186/s12885-015-1831-7

**Published:** 2015-11-06

**Authors:** Tomoko Tanaka, Katsushi Hiramatsu, Takuto Nosaka, Yasushi Saito, Tatsushi Naito, Kazuto Takahashi, Kazuya Ofuji, Hidetaka Matsuda, Masahiro Ohtani, Tomoyuki Nemoto, Hiroyuki Suto, Tatsuya Yamamoto, Hirohiko Kimura, Yasunari Nakamoto

**Affiliations:** Second Department of Internal Medicine, Faculty of Medical Sciences, University of Fukui, 23-3 Matsuoka Shimoaizuki, Eiheiji-cho, Yoshida-gun, Fukui, 910-1193 Japan; Department of Radiology, Faculty of Medical Sciences, University of Fukui, Fukui, Japan

**Keywords:** Metastasis to pituitary gland, Hepatocellular carcinoma, Hypopituitarism

## Abstract

**Background:**

Metastasis to the pituitary gland is extremely rare and is often detected incidentally by symptoms associated with endocrine dysfunction. Breast and lung cancer are the most common primary metastasizing to pituitary gland. Metastasis from hepatocellular carcinoma to the pituitary gland is extremely rare, with only 10 cases having been previously reported. We present here the first case of pituitary metastasis of hepatocellular carcinoma presenting with panhypopituitarism diagnosed by magnetic resonance imaging.

**Case presentation:**

We report the case of an 80-year-old Japanese woman who presented with the sudden onset of hypotension and bradycardia after having previously been diagnosed with hepatocellular carcinoma. Based on low levels of pituitary hormones, she was diagnosed with panhypopituitarism caused by metastasis of the hepatocellular carcinoma to the pituitary gland. Magnetic resonance imaging with arterial spin-labeling was effective in the differential diagnosis of the intrasellar tumor. The patient died despite hormone replacement therapy because of hypovolemic shock.

**Conclusion:**

Metastasis to the pituitary gland causes various non-specific symptoms, so it is difficult to diagnose. The present case emphasizes the importance of diagnostic imaging in identifying these metastases. Clinicians should consider the possibility of pituitary metastasis in patients with malignant tumors who demonstrate hypopituitarism.

## Background

Intracranial metastasis to the pituitary gland is rare. The reported incidence of pituitary metastasis is 0.87 % and 1.9 % of all intracranial metastases and autopsied cancer patients, respectively. Breast and lung cancer are the most common primary carcinomas causing pituitary metastasis [1]. Metastasis from hepatocellular carcinoma (HCC) to the pituitary gland is extremely rare, with only 10 cases having been previously described [1–9]. Here, we report a case of pituitary metastasis from HCC in which the patient presented with hypotension and bradycardia. In addition, we highlight the use of magnetic resonance imaging (MRI) for the differential diagnosis of the intrasellar tumor.

## Case presentation

In November 2011, an 80-year-old woman exhibited sudden-onset anorexia accompanied by hypotension and bradycardia. She had previously been treated for hepatitis C virus-related cirrhosis and HCC in August 2002. The patient suffered multiple relapses of HCC and had undergone four radiofrequency ablation cycles and two transcatheter arterial chemoembolization cycles. Routine laboratory tests detected a mild elevation of serum aspartate aminotransferase to 81 IU/L (normal level, <34 IU/L). An additional endocrinological work-up detected thyroid hormone, cortisol, and adrenocorticotropic hormone insufficiencies, with normal levels of thyroid stimulation hormone (Table 1). These results were indicative of hypopituitarism.

**Table 1:** Basal endocrine evaluation

Hormone	Level measured	Normal levels
GH	0.360 ng/mL	0.010–3.607 ng/mL
PRL	6.83 ng/mL	<12.3 ng/mL
TSH	1.288 μIU/mL	0.350–4.940 μIU/mL
FT3	1.78 pg/mL	1.71–3.71 pg/mL
FT4	**<0.40 ng/dL**	0.70–1.48 ng/dL
ACTH	**1.4 pg/mL**	7.2–63.3 pg/mL
Cortisol	**3.1 μg/dL**	4.0–19.3 μg/dL
Aldosterone	**10 pg/mL**	36–240 pg/mL
Plasma renin activity	0.5 ng/mL	0.2–3.9 ng/mL
ADH	**0.27 pg/mL**	0.3–4.2 pg/mL
LH	**<0.10 mIU/mL**	7.5–56.2 mIU/mL
FSH	**0.27 mIU/mL**	9.2–124.7 mIU/mL

Bold text indicates abnormal data

GH: growth hormone, PRL: prolactin, TSH: thyroid-stimulating hormone, FT3: free triiodothyronine, FT4: free thyroxine, ACTH: adrenocorticotropic hormone, ADH: antidiuretic hormone, LH: luteinizing hormone, FSH: follicle-stimulating hormone

**Fig. 1:**
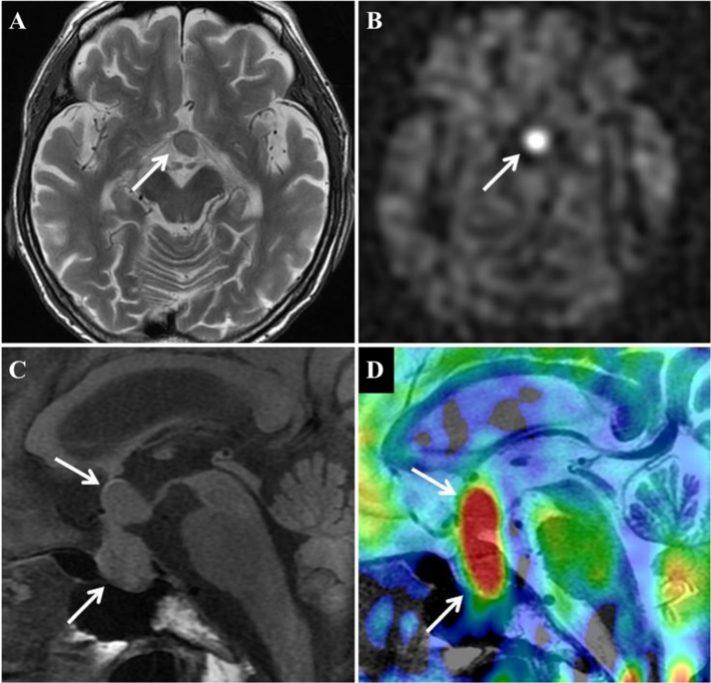
Magnetic resonance images of the patient on admission. (**a**) Axial T2-weighted image showing a tumor located in supra-sellar region (arrow). The tumor is imaged as same intensity as gray matter. (**b**) Arterial spin labeling (ASL) perfusion weighted image corresponding to the section of (**a**). The tumor is revealed as bright hyper-intensity mass (arrow), suggesting elevated blood flow. (**c**) Sagittal T1-weighted pre-contrast image. The tumor is located in pituitary fossa and had spread across the suprasellar region (arrows). (**d**) ASL fusion image onto a T2-weighted image. The fusion image demonstrates high blood flow signal exactly corresponds to the tumor mass (arrows).

MRI of the brain revealed a tumor measuring 13 mm × 13 mm in the sella turcica, which had spread across the suprasellar region (Fig. 1a) and was likely the cause of the panhypopituitarism. Magnetic resonance perfusion with arterial spin-labeling (ASL) indicated that the tumor had hyperperfusion nature (Fig. 1b). This finding was compatible with pituitary metastasis, with HCC as the primary lesion. Subsequent computed tomography (CT) scans of the thorax, abdomen, and pelvis identified no other primary lesions. Finally, a clinical diagnosis of HCC metastasis to the pituitary gland causing panhypopituitarism was made.

Steroid and thyroid hormone replacement therapy was used to treat the patient’s panhypopituitarism, resulting in stabilization of her blood pressure and pulse. However, diabetes insipidus (DI) developed five days into the replacement therapy, and the patient’s daily output of urine was more than 2.5 L. Urine output decreased only temporarily following treatment with desmopressin, but increased to 6.0 L/day one week into treatment. Two weeks after the initial diagnosis of pituitary metastasis, MRI revealed enlargement and subsequent hemorrhaging of the metastatic pituitary tumor.

**Fig. 2:**
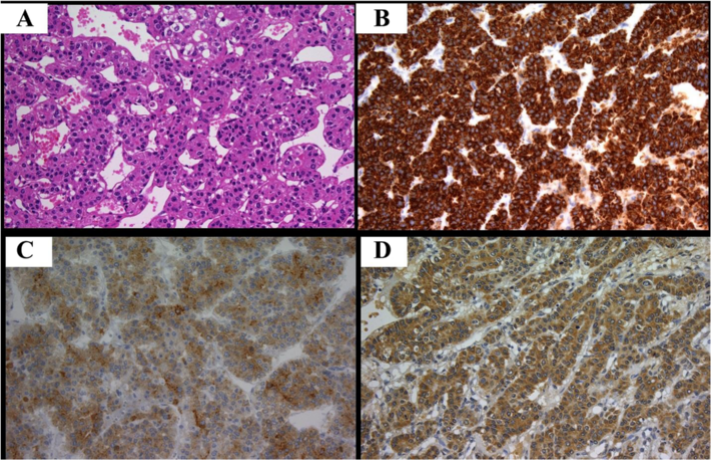
Histological tumor specimens (magnification × 400). (**a**) Hematoxylin and eosin staining of the pituitary tumor demonstrating a tumor with cord-like structure. Immunohistochemical expression of (**b**) hepatocytes, (**c**) glypican-3, and (**d**) α-fetoprotein.

In January 2012, the patient died of hypovolemic shock. Postmortem examination of the pituitary tumor revealed tumoral hepatocytes in a thick trabecular pattern, the typical appearance of well differentiated HCC (Fig. 2a). The tumor was immunopositive for hepatocytes, α-fetoprotein, and glypican-3 (Fig. 2, b-d). These findings were compatible with a diagnosis of HCC metastasis to the pituitary gland.

## Discussion

As noted above, metastasis to the pituitary gland is extremely rare. The incidence of pituitary metastasis is less than 1 % among all patients with intracranial metastases [1]. A review of the literature revealed that breast and lung cancers are the most common primary tumors to metastasize to the pituitary gland. The prognosis of pituitary metastasis is poor and is related to the histological subtype and stage of the primary malignancy rather than to the presence of the metastasis itself [1].

The differential diagnosis of intrasellar tumors requires the exclusion of such diseases as hypophyseal adenoma, craniopharyngioma, meningioma, Rathke’s cleft cyst, and aneurysm. Fassett et al. reported that differentiation of a pituitary metastasis from other pituitary tumors based on neuroimaging alone can be difficult, although certain features, such as thickening of the pituitary stalk, invasion of the cavernous sinus, and sclerosis of the surrounding sella turcica, can indicate metastasis [10]. On the other hand, Post points out in his recent commentary that imaging techniques, such as MRI or CT, can distinguish metastatic disease if the pituitary gland is visually separate from the tumor or if excessive bone destruction is evident [11]. In the current case, although we did not observe these specific features of metastatic disease, magnetic resonance perfusion with ASL allowed us to differentially diagnose the pituitary metastasis.

ASL is a unique perfusion imaging method, especially for the brain, that can generate images without injection of contrast material. Its clinical utility has been established in several cerebral conditions such as brain tumors, infarction, and vascular lesions [12–14]. In our case, ASL clearly showed hyper-perfusion nature of the tumor, indicative of a metastatic tumor rather than pituitary macro adenoma, which is more frequent but rarely exhibit such blood flow, though other supra-sellar tumors such as meningioma or craniopharyngioma may also be revealed as hyperperfusion on ASL image.

The patient exhibited a sudden onset of hypotension with bradycardia, symptoms which were suspected to be the result of panhypopituitarism. This was later confirmed by a blood test. On the other hand, the most common symptom of pituitary metastasis is DI [6–8, 15], because the posterior lobe receives direct blood flow from systemic circulation [9, 15]. However, Peppa et al. described that the symptoms of metastasis to the pituitary gland are not specific and include fatigue, weight loss, dizziness, nausea, and vomiting. Therefore, symptoms of pituitary metastasis are often mistaken for those of cancer cachexia [16]. These data suggest that metastasis to the pituitary gland should be suspected when a patient with advanced malignancy shows unaccountable, non-specific symptoms.

Distinguishing metastasis to the pituitary gland from metastasis to the skull base (sphenoid sinus, cavernous sinus, and sella turcica) is very difficult because of the close proximity of these regions [15]. To establish a diagnosis, pathological confirmation is required. In general, bone metastasis from HCC is estimated to occur in 2-16 % of all HCC patients and may often spread to the vertebrae, ribs, pelvis, and long bones [17]. In the current case, metastasis of HCC to the skull base could not be excluded during the patient’s lifetime, but metastasis to the pituitary gland was definitively diagnosed by histological findings in the postmortem examination.

For the treatment of metastatic pituitary tumors, Morita et al. reported that there were no significant differences in survival times between surgical and nonsurgical treatments [9]. The vascularity of metastatic tumors is extremely rich; therefore, surgery is sometimes performed not only for palliation, but also for prevention of hemorrhage [15]. Additionally, surgery improves the quality of life and provides pathologic confirmation of the disease [11]. Before surgery and biopsy, it is advantageous for ASL image to obtain how much of vasculature the tumor has and to assess the risk of bleeding at the surgical treatment. In the current case, surgery was not performed because the patient was in the terminal stage of advanced HCC. However, if the growth of HCC is well controlled, surgery is an attractive choice.

## Conclusions

We reported a case of HCC metastasis to the pituitary gland presenting with panhypopituitarism. The present case emphasizes the importance of diagnostic imaging in identifying these metastases. Metastasis to the pituitary gland causes various non-specific symptoms. It is important to consider a metastasis to the pituitary gland when an HCC patient exhibits nonspecific symptoms associated with panhypopituitarism.

## Consent

Written informed consent was obtained from the guardians of the patient for publication of this case report and any accompanying images. A copy of the written consent is available for review by the Editor of this journal.

## Abbreviations

HCC: hepatocellular carcinoma
MRI: magnetic resonance imaging
ASL: arterial spin labeling
CT: computed tomography
DI: diabetes insipidus
